# Clinical repercussions of Glanders (*Burkholderia mallei* infection) in a Brazilian child: a case report

**DOI:** 10.1590/0037-8682-0054-2020

**Published:** 2020-06-22

**Authors:** Eusébio Lino dos Santos, Juliane de Carvalho Rocha Moura, Bruna Karoline Pinheiro França Protásio, Vanise Aragão Santos Parente, Maria Helena Neves Dorea Veiga

**Affiliations:** 1 Universidade Tiradentes, Departamento de Medicina, Aracaju, SE, Brasil.; 2 Hospital de Urgência de Sergipe, Aracaju, SE, Brasil.

**Keywords:** Glanders, Zoonosis, Burkholderia mallei

## Abstract

Glanders is a relatively unknown zoonotic disease caused by *Burkholderia mallei.* This bacterium affect solipeds and humans, and can be used as a biological warfare. Glanders is characterized as an occupational disease. We report the case of an 11-year-old boy who was presented to an emergency department with chest pain and dyspnea. He evolved into septic shock, pneumonia, and multiple abscesses. *B. mallei* was found in the exudate culture. Human infection is rare and difficult to confirm. The knowledge on glanders is important for differential diagnosis from other serious illnesses causing pneumonia and multiple abscesses.

## INTRODUCTION

Glanders is caused by *Burkholderia mallei*. The Greeks first described it in 450-425 BC and then by the Romans in 400-500 AD^1^. It is a zoonotic disease. It affects the horses causing a chronic disease, and affects mules and donkeys causing an acute disease^2^. *B. mallei* also causes an occupational disease in human; it infects individuals who have frequent and close contact with infected animals such as veterinarians, grooms, and farmers or those who have laboratorial exposure, such as microbiologists. In such cases, infection mainly occurs through the contamination of wounds or inhalation^3^.

This disease is more common where equine glanders is endemic, such as Eastern Europe, and the Middlewest and Southeast Asia^4^. However, sporadic cases are present in the Western world^5^ Although a human epidemic has not been reported, isolated outbreaks in human populations and the deliberate use of *B. mallei* as a biological weapon have been documented^2^.

*B. mallei* is currently listed on the Select Agents and Toxins list compiled by the US Centers for Disease Control and Prevention. It has also been determined to have the potential to pose a severe threat to human and animal health^6^.

Glanders is relatively unknown in Brazil with few epidemiological data; its clinical symptoms in humans are non-specific. Therefore, we describe the first reported case of human Glanders in a child from the Northeast of Brazil, and discuss some of the important facts in the disease history.

## CASE REPORT

An 11-year-old boy from the outskirts of Aracaju, previously healthy, complained of strong chest pain that had started four days before his admission to the hospital. He was in constant close contact with families who owned horses. 

**FIGURE 1:**
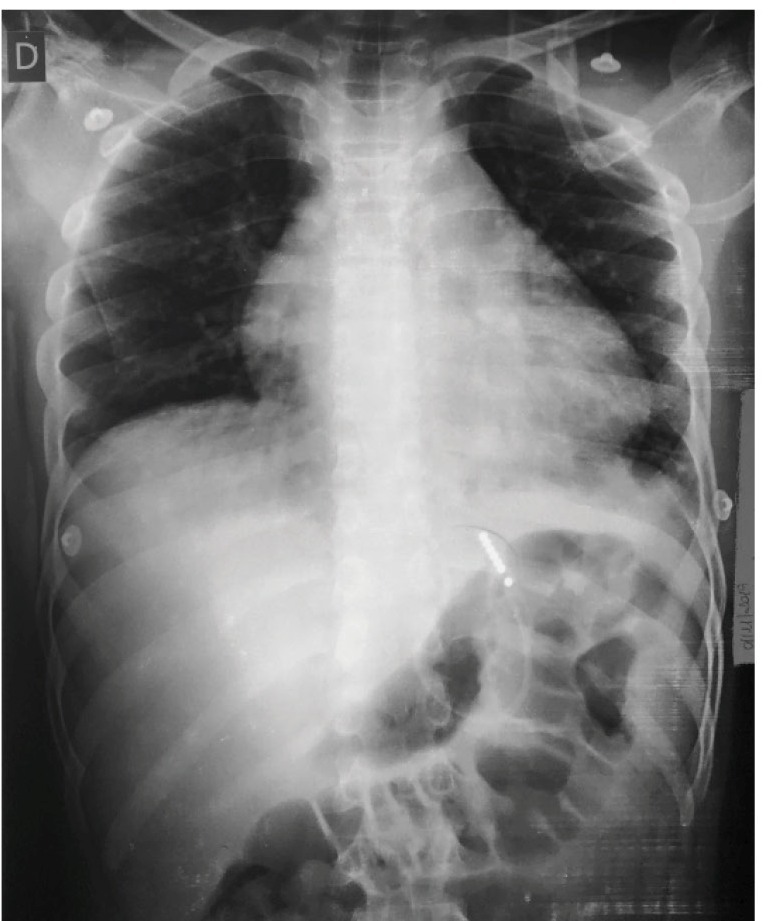
Chest radiography suggestive of mediastinal widening.

At his first visit, chest radiography was performed but nothing was identified. The patient received bronchodilators and was sent home. Because his chest pain worsened, associated with the onset of dyspnea and fever, he sought emergency care again and was promptly admitted. An abrasion was observed in his left knee. Chest radiography was performed again and a mediastinal widening was observed (Figure 1). This suggested the hypothesis of increased cardiac area due to infectious pericarditis. Oxacilin and gentamicina were prescribed as initial treatment.

**FIGURE 2:**
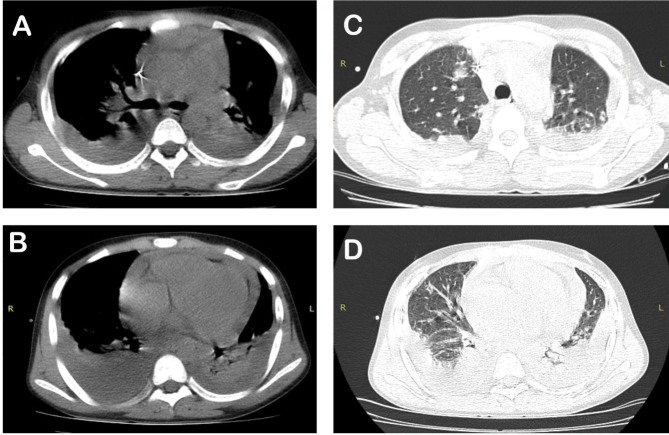
(A) Hilar lymphadenomegaly associated with segmental compressive atelectasis on the left side. (B) Bilateral pleural effusion with basal consolidations and pericardial effusion. (C) Peripheral nodular opacities in the pulmonary apices, some subpleural confluent forming consolidations. (D) Bilateral ground-glass opacities and consolidations associated with inflammation in both lungs.

The patient underwent significant clinical worsening; in addition to shock (was given vasoactive drugs), he experienced pericardial effusion and pneumonia with bilateral pleural effusion, (observed using chest computed tomography) (Figure 2).

*Staphylococcus haemolyticus* with multisensitive pattern on antibiotic susceptibility testing was found in the blood of the patient; therefore, treatment with oxacillin and gentamicin was continued. However, because the fever persisted and multiple abscesses appeared on his trunk, drainage was performed in the operating room, and the exudate was collected to prepare the bacterial culture. Subsequently, *Burkholderia mallei* was identified. Therefore, an intensive phase of the treatment was initiated with intravenously (IV) meropenem for 21 days, along with an eradication phase treatment with trimethoprim/sulfamethoxazole (TMP-SMX) IV for 21 days. Besides, TMP-SMX was given orally for an additional 9 weeks. At the end of the 12 weeks of treatment, the patient showed significant clinical improvement.

## DISCUSSION

Glanders is a rare disease and case reports in humans are scarce. This infection is caused by *B. mallei,* a Gram-negative, aerobic, spore-forming, nonmotile, facultative intracellular bacteria^2^ ^,^ ^3^. It is one of the few agents that has been used deliberately to infect human and other animals. Because of the lethal and contagious nature of the disease, *B. mallei* was considered an ideal agent for biological warfare and was used for by Germany in World War I^7^ ^,^ ^8^.

In humans, it is primarily an occupational disease, affecting individuals in close contact with infected animals such as veterinarians, grooms and farmers, or those with laboratory exposure^6^. Zoonotic transmission appears to be uncommon, even in a frequent and close contact. Infection generally occurs via the respiratory tract or direct invasion of punctured skin after contact with infected animals^9^. The course of infection depends on the route of exposure; direct contact with the skin can lead to a localized cutaneous infection, while inhalation of aerosol or dust containing *B. mallei* can lead to septicemic or pulmonary infection^2^. The acute form of the disease has a typical incubation period of 1-14 days^1^.

Clinical manifestations include initial onset of fever, rigors and malaise, respiratory difficulties culminating in a rapid onset of pneumonia, bacteremia, pustular skin lesions, and development of multiple abscess-forming nodules, known as farcy^9^. Septicemic glanders may produce numerous signs consistent with a highly pathogenic bacterial septicemia^10^.

The diagnosis is usually difficult and cannot be based on clinical evaluation alone because glanders might cause a wide variety of non-unique clinical manifestations^6^. Glanders can be diagnosed when the causative organism is isolated and correctly identified. It is often difficult to find *B. mallei*, even in acute abscesses, and blood cultures are frequently negative until the terminal stages of the disease^10^.

There are some data on the efficacy of antibiotics for the treatment of human glanders; however, no Food and Drug Administration (FDA) approved treatment is available. The current treatment option includes a mixed antibiotic regimen that is often only partially effective. Treatment is prolonged consisting of both intensive intravenous (IV) and oral eradication therapies. IV therapy includes imipenem, and meropenem or ceftazidime with or without TMP-SMX, and should be continued for a minimum of 10 days. The oral antibiotic therapy is TMP-SMX with or without doxycycline and may run 12 weeks to as long 12 months of duration^1^ ^,^ ^10^. Surgical drainage of abscesses may be required in some cases^3^. The mortality rate for pulmonary and septicemic forms of the disease has been reported to be 90-95% without treatment, while mortality rate with treatment is 40% and 50%, respectively^1^. Currently there is no vaccine available for preventing *B. mallei* infection.

Glanders is probably under recognized and underreported. This case demonstrates the difficulties that the medical team may face in identifying *B. mallei* infection. Considering the high mortality rate when glanders remain untreated, the occupational or contact history along with the presence of sepsis, multiple abscesses, and pneumonia should prompt the consideration of *B. mallei* infection.
